# Differences in the Gene Expression Profiles of Haemocytes from Schistosome-Susceptible and -Resistant *Biomphalaria glabrata* Exposed to *Schistosoma mansoni* Excretory-Secretory Products

**DOI:** 10.1371/journal.pone.0093215

**Published:** 2014-03-24

**Authors:** Zahida Zahoor, Anne E. Lockyer, Angela J. Davies, Ruth S. Kirk, Aidan M. Emery, David Rollinson, Catherine S. Jones, Leslie R. Noble, Anthony J. Walker

**Affiliations:** 1 Molecular Parasitology Laboratory, School of Life Science, Kingston University, Kingston upon Thames, Surrey, United Kingdom; 2 Wolfson Wellcome Biomedical Laboratory, Natural History Museum, London, United Kingdom; 3 Institute of Biological and Environmental Sciences, School of Biological Sciences, Aberdeen University, Aberdeen, United Kingdom; Queensland Institute of Medical Research, Australia

## Abstract

During its life cycle, the helminth parasite *Schistosoma mansoni* uses the freshwater snail *Biomphalaria glabrata* as an intermediate host to reproduce asexually generating cercariae for infection of the human definitive host. Following invasion of the snail, the parasite develops from a miracidium to a mother sporocyst and releases excretory-secretory products (ESPs) that likely influence the outcome of host infection. To better understand molecular interactions between these ESPs and the host snail defence system, we determined gene expression profiles of haemocytes from *S. mansoni*-resistant or -susceptible strains of *B. glabrata* exposed *in vitro* to *S. mansoni* ESPs (20 μg/ml) for 1 h, using a 5K *B. glabrata* cDNA microarray. Ninety-eight genes were found differentially expressed between haemocytes from the two snail strains, 57 resistant specific and 41 susceptible specific, 60 of which had no known homologue in GenBank. Known differentially expressed resistant-snail genes included the nuclear factor kappa B subunit Relish, elongation factor 1α, 40S ribosomal protein S9, and matrilin; known susceptible-snail specific genes included cathepsins D and L, and theromacin. Comparative analysis with other gene expression studies revealed 38 of the 98 identified genes to be uniquely differentially expressed in haemocytes in the presence of ESPs, thus identifying for the first time schistosome ESPs as important molecules that influence global snail host-defence cell gene expression profiles. Such immunomodulation may benefit the schistosome, enabling its survival and successful development in the snail host.

## Introduction

The parasite *Schistosoma mansoni* causes the neglected tropical disease human intestinal schistosomiasis, and has a complex life cycle that involves a freshwater snail intermediate host, a human definitive host, and free-living motile stages that enable movement between hosts [1]. *Biomphalaria glabrata* is the main intermediate host for *S. mansoni* in South America and like other snails it possesses a potent internal defence system enabling protection against pathogens [2], which however the parasite is able to overcome in a compatible snail host. Haemocytes, macrophage-like defence cells, are the main effectors of the internal defence response in snails and are capable of performing multiple defence reactions including phagocytosis [3], [4], encapsulation, and nitric oxide (NO) [5] and hydrogen peroxide (H_2_O_2_) [6], [7] production. Over the last two decades, the *B. glabrata*-*S. mansoni* model has proved invaluable for studying snail-schistosome interactions and coevolution, particularly because of its biomedical significance and because snail strains are available that are resistant or susceptible to *S. mansoni* infection [8]–[10].

When a *S. mansoni* egg hatches upon contact with freshwater, the free-living miracidium emerges and swims in search of a compatible host snail, which it will penetrate. The parasite then rapidly transforms into a post-miracidium losing its ciliated plates, and develops into the asexually-reproducing mother sporocyst that produces daughter sporocysts, which in turn produce human-infective cercariae [1]. During the early post-embryonic development of miracidia to mother sporocysts various molecules termed excretory-secretory products (ESPs; [11]) are released from the parasite; some of these are likely to be from the penetration glands and surface structures whereas others are metabolic by-products released from the excretory pore. ESPs generally comprise molecules such as cysteine proteases, protease inhibitors, heat shock proteins (HSPs), mucins, glycolytic enzymes, anti-oxidant enzymes, and ion-binding proteins [12]–[14]. *In vitro* studies have shown that *S. mansoni* ESPs affect *B. glabrata* haemocyte defence responses such as motility [15], and NO [16] and superoxide production [17]. We have recently shown that *S. mansoni* ESPs suppress signalling by extracellular signal-regulated kinase (ERK) in haemocytes from *S. mansoni*-susceptible *B. glabrata* [18] and that such suppression likely affects HSP70 [19] and NO [16] levels in a dose dependent fashion. This supports the interference theory [20] (reviewed by [21]) whereby the parasite is able to suppress the host response to enable it to establish an infection. Strikingly, ESPs did not affect ERK signalling in haemocytes from a *S. mansoni*-refractory *B. glabrata* strain [18] demonstrating that haemocytes from a resistant strain reacted differently to the presence of parasite ESPs, indicating a different response at the molecular level, which may be responsible for the outcome of infection. Thus, by producing ESPs schistosomes seem able to modulate the defence responses of host snail haemocytes to facilitate parasite survival. However, the extent to which ESPs modulate global gene expression of haemocytes remains an important and unanswered question.

Arrays developed for determining gene expression patterns in snails include oligo arrays and cDNA microarrays [22]–[24]. A 5K *B. glabrata* cDNA microarray was recently developed [25] using sequences obtained from differential display [26] and suppression subtractive hybridization (SSH) [10] gene expression projects focusing on schistosome resistance/susceptibility, and from ORESTES-derived expressed sequence tags (ESTs) from different snail tissues including haemocytes [27]. This 5K array was used to evaluate differential gene expression in haemocytes from schistosome-resistant and -susceptible *B. glabrata* in response to a two hour *in vivo* infection by *S. mansoni* [25]. In the current study, we have employed this *B. glabrata* cDNA microarray to explore the hypothesis that haemocytes from these snail strains respond differentially to *S. mansoni* ESPs released during miracidium-to-mother sporocyst development. Ninety-eight genes were found differentially expressed in these haemocytes in the presence of ESPs and comparative analysis with previous studies revealed that 38 were unique ESP-mediated responses. These findings highlight that the ESPs may play an important part in defining the ability of *S. mansoni* to sustain an infection in *B. glabrata*.

## Methods

### Ethics Statement

Laboratory animal use was within a designated facility regulated under the UK animals (Scientific Procedures) Act, 1986, complying with all requirements therein. The Natural History Museum (NHM) Ethical Review Board approved experiments involving mice and work was carried out under Home Office project licence 70/6834.

### Snails

Two snail strains were used, one resistant to *S. mansoni* infection (NHM accession number 3017) originally derived from BS90 [28], and one susceptible (NHM1742). Snails were maintained at 26°C with a 12:12 h light:dark cycle in water that had been filtered through a Brimak/carbon filtration unit (Silverline, Winkleigh, UK) and were fed fresh round lettuce *ad libitum*.

### Preparation of *S. mansoni* ESPs

*In vitro* transformation of miracidia to mother sporocysts [29] and the preparation of resultant ESPs have been detailed previously [16], [18], [19]. Briefly, *S. mansoni* (Belo Horizonte strain) eggs were collected and left to hatch in spring water (Evian) for 3 h under light. Miracidia were then collected, washed and concentrated using a Stericup HV filter with a 0.45 μm membrane (Millipore, Watford, UK) and were placed in 25 cm^2^ vented tissue culture flasks in Chernin's balanced salt solution (CBSS) [30] containing glucose and trehalose (1 mg/ml each), penicillin and streptomycin (100 U/ml of each; Sigma, Poole, UK). Flasks were placed in the dark in an incubator at 26°C for 36–40 h and transformation monitored using an inverted light microscope. The CBSS containing ESPs was then concentrated approximately 20X at 4°C using Vivapore 10 ml concentrators (7,500 MW cut-off; Vivascience, Sartorius, Epsom, UK). Protein concentration of the ESPs was then determined against bovine serum albumin standards using a NanoOrange fluorescence-based protein assay kit (Molecular Probes, Leiden, Netherlands) and a Fluorstar Optima microplate spectrofluorimeter (BMG Labtech, Aylesbury, UK). In general, using the above approaches, we collect approximately 1 μg ESPs from ∼100 transforming miracidia. The ESP solution was then divided into aliquots and stored at −20°C until used.

### Treatment of Haemocytes and RNA Extraction

Haemolymph (1 ml) from 35 – 40 *S. mansoni*-susceptible or -resistant *B. glabrata* was extracted using head-foot retraction and was pooled on ice in CBSS (2 parts haemolymph: 1 part CBSS) supplemented with glucose and trehalose (1 mg/ml each). Haemocyte monolayers were prepared by allowing haemocytes to adhere to individual wells of a 12-well flat bottom cell culture plate (Corning Costar, Schiphol-Rijk, The Netherlands) for 30 min at room temperature. The monolayers were then washed three times each with 500 μl CBSS and were equilibrated for a further 30 min before being challenged with 20 μg/ml *S. mansoni* ESPs for 1 h. On average we recovered ∼25 μl haemolymph per snail; therefore haemocytes in this volume would be exposed to approximately 0.5 μg ESPs [25 μl/(20 μg ESPs/1000 μl)] which equates to ESPs from ∼50 transforming miracidia. The CBSS containing ESPs was removed and RNA extracted from haemocytes using Trizol (Invitogen, Paisley, UK) according to the manufacturer's instructions. RNA was subsequently treated with DNAse (Promega, Southampton, UK) followed by ethanol precipitation and resuspension in nuclease free water. Quantities of total RNA as well as its purity were estimated using a NanoDrop spectrophotometer.

### Microarray Hybridization

Labelled cDNA was prepared for microarray hybridization as described previously [25]. Briefly, cDNA was synthesized from 1 μg total RNA using the SMART PCR [31] cDNA synthesis kit (Clontech-Takara Bio, Europe, Saint-Germain-en-Laye, France) according to the manufacturer's instructions and each sample was labelled with both Cy3 dCTP or Cy5 dCTP (GE Healthcare, Amersham, UK) in 2 separate reactions (dye swap replicates) using the BioPrime DNA labeling system (Invitrogen, Paisley, UK). The custom 5K *B. glabrata* cDNA microarrays [25] (printed at the Microarray facility at Department of Pathology, University of Cambridge, UK) include cDNA clones from open reading expressed sequence tags (ORESTES) libraries [27] and suppression subtractive hybridization (SSH) libraries [10]. Four microarray hybridizations (technical replicates) were performed with resistant and susceptible derived material as previously described [24], [25].

### Microarray Scanning and Analysis

Microarray slides (n = 4) were scanned sequentially for each Cy dye, at 10 μm resolution using an Axon GenePix 4100A scanner (Molecular Devices (UK) Ltd, Wokingham, UK). Photo multiplier tube values were adjusted to give an average intensity ratio between channels of approximately 1. Spot finding and intensity analysis was carried out using GenePix Pro 5.0. Data from these microarray experiments have been deposited with ArrayExpress (www.ebi.ac.uk/arrayexpress) under the accession number E-MTAB-2135. The identification of differentially expressed genes was performed as previously described [24]. Briefly, mean pixel intensity (Feature(wavelength)-Background(wavelength)) was normalized using intensity-dependent Lowess normalization for each feature [32] and consistency within each array was assessed by comparing normalized mean pixel intensity ratios (Cy3/Cy5) for each duplicate feature (Acuity 4.0). Poor quality spots and low intensity data were removed. For each array the mean intensity values for pGem (vector) controls were calculated to give a background level of hybridization and only features with intensities greater than the mean plus one standard deviation threshold in either channel (Cy3 or Cy5) were retained. Consistency between array replicates was assessed by comparing mean (from the two duplicates) intensity ratios for each clone. The mean and standard deviation of the remaining data, excluding SSH clones, were used to calculate 95% confidence limits for the normalized intensities for each array and those features which showed differential expression outside this 95% level marked. Genes that passed the 95% confidence level in 3 or 4 of the arrays were considered to demonstrate differential expression. These genes were then clustered on the basis of their sequence, using SeqTools (http://www.seqtool.dk) to identify unique sequences. Gene ontology (GO) functions were assigned using Blast2go (http://www.blast2go) based on BLASTX homologies.

## Results

### Expression Profiling by Microarray

The 5K microarray was used here to compare directly gene expression in haemocytes derived from schistosome-resistant and -susceptible *B. glabrata* when treated with *S. mansoni* ESPs produced during miracidia to mother sporocyst development. Differential expression of haemocyte genes determined using this *B. glabrata* cDNA microarray has been confirmed previously in our laboratory by qPCR and the array was found to deliver robust results [24]. Haemocytes were exposed to ESPs for 1 h as this duration was considered important in terms of early haemocyte defence responses. Because only very low amounts of RNA are available in haemocytes, independent SMART amplifications and labelling reactions were carried out and four technical replicate hybridizations performed, as detailed in our previous array studies [24]. As total ESPs were derived from miracidia from eggs present in the livers of several mice [16], [18], [19], and because haemocyte pools were made from 35 – 40 snails of each snail strain, the results represent those from populations of miracidia and snails. Moreover, the ESPs were prepared in such a way that only those molecules possessing a molecular weight >7500 were present in the preparation used for haemocyte exposures; therefore ciliary epidermal plates were excluded. Following 1 h ESP exposure, 57 resistant-specific and 41 susceptible-specific genes were identified from the snail haemocytes (Table S1). All of these 98 genes displayed between ∼1.9- and ∼5.3-fold (mean of 4 arrays) difference in expression between haemocytes from the resistant and susceptible snails. Of the identified differentially expressed genes, 36% were derived from SSH, 36% from ESTs and 28% from ORESTES. Certain differentially expressed genes were represented by two or more sequences on the array. For example, elongation factor-2 was represented by two SSH sequences (EW997555, EW997067), whereas matrilin (CK656770, CK988725) and paramyosin (CK656849, CO870407, EW996929, CO870250) were from ORESTES and EST libraries, and SSH and ORESTES libraries, respectively (Table S1).

### Annotation of Differentially Expressed Sequences

**Figure 1:**
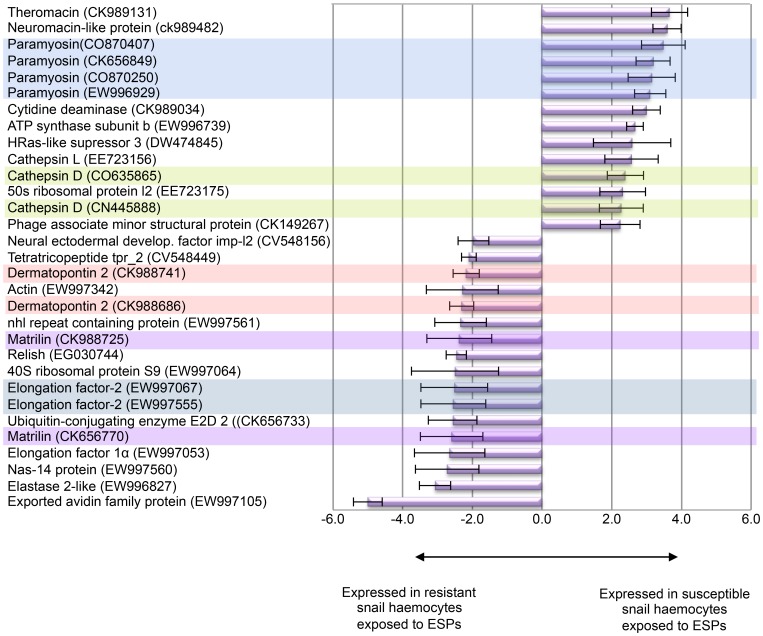
Genes differentially expressed between haemocytes from schistosome-resistant and -susceptible *B. glabrata* exposed to *S. mansoni* ESPs. Only genes with known homologues are shown. Each bar represents the mean (±SD) normalised expression for the identified differentially expressed gene (p≤0.05, n = 4). A positive value indicates the gene is expressed in susceptible snail haemocytes whereas a negative value indicates the gene is expressed in resistant snail haemocytes. Multiple genes matching the same protein are shaded in a similar colour.

Using BlastX, 61 of the 98 identified differentially expressed genes did not match others in GenBank and were therefore categorized as having unknown function; an additional six genes matched hypothetical proteins from a range of lower organisms and *Xenopus* (Table S1). The remaining 31 genes corresponded to 24 known proteins from a variety of organisms, of these 14 were specific to resistant snail haemocytes and 10 to susceptible (Fig. 1). Gene cluster analysis of the 98 genes mapped 14 into six different clusters, with two clusters each containing three unknowns (EW996998, EW997485, DY523254; EW997449, EW996825, EW997106) all of which were resistant-specific (Table S1). The remaining four clusters, each of which contained two genes, comprised three resistant-specific groups (CK656770, CK988725: matrilin; EW997555, EW997067: elongation factor-2; EW997374, DY523259: unknown) and one susceptible-specific group (CO870250, CO870407: paramyosin) (Table S1).

**Figure 2:**
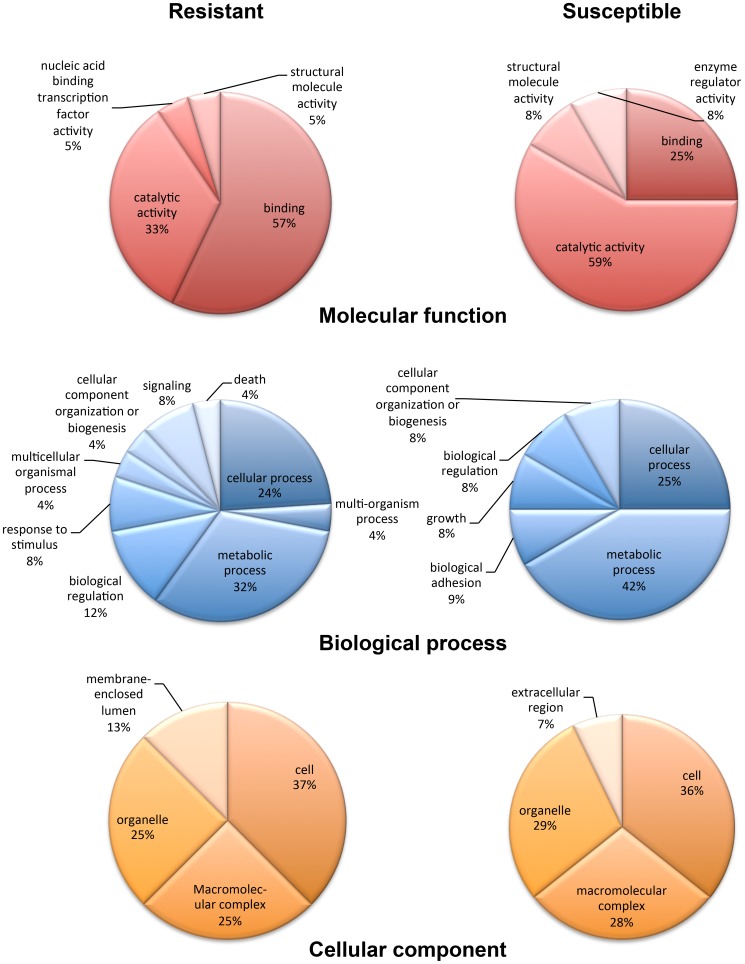
Gene ontologies for differentially expressed genes that possessed GO matches identified in haemocytes from *S. mansoni*-resistant or -susceptible *B. glabrata*. Categories are grouped according to molecular function, biological process, and cellular component. Sequences that were not assigned an annotation are not represented and each individual sequence may have more than one assignment.

Matched genes associated with immune mechanisms included Relish (EG030744) that was differentially expressed in resistant snail haemocytes by approximately 2.5-fold (mean of 4 arrays). The antimicrobial peptide theromacin (CK989131) was the greatest (3.7-fold) differentially expressed gene with a BlastX match in susceptible snail haemocytes, whereas exported avidin family protein (∼5-fold; EW997105) was the most differentially expressed BlastX-matched resistant-specific gene (Fig. 1). To establish grouped gene function the matched sequences were assigned GO categories in the context of snail phenotype (Fig. 2). In resistant snail haemocytes there was a preponderance of genes associated with the molecular function ‘binding’ (57%; 12 genes) (nucleic acid, protein, ion, or nucleotide binding) when compared to susceptible snail haemocytes (25%; 3 genes) with the latter displaying a higher overall proportion of genes related to catalytic activity (59% v's 33%). Genes for ‘metabolic processes’ (biological process) were overrepresented (5 out of 12 genes; 42%) in the susceptible strain when compared to the proportion seen in the resistant (8 out of 25 genes; 32%) (Fig. 2). Four (8%) cell ‘signalling’/‘response to stimulus’ genes (biological process) were overexpressed in the resistant snail haemocytes with none in the susceptible. Finally, GO analysis of cellular component revealed no major differences between strains although genes associated with ‘extracellular region’ were only overexpressed in the susceptible snail haemocytes and those associated with ‘membrane enclosed lumen’ were only overexpressed in haemocytes from the resistant strain.

### Comparative Gene Expression Analysis

**Figure 3:**
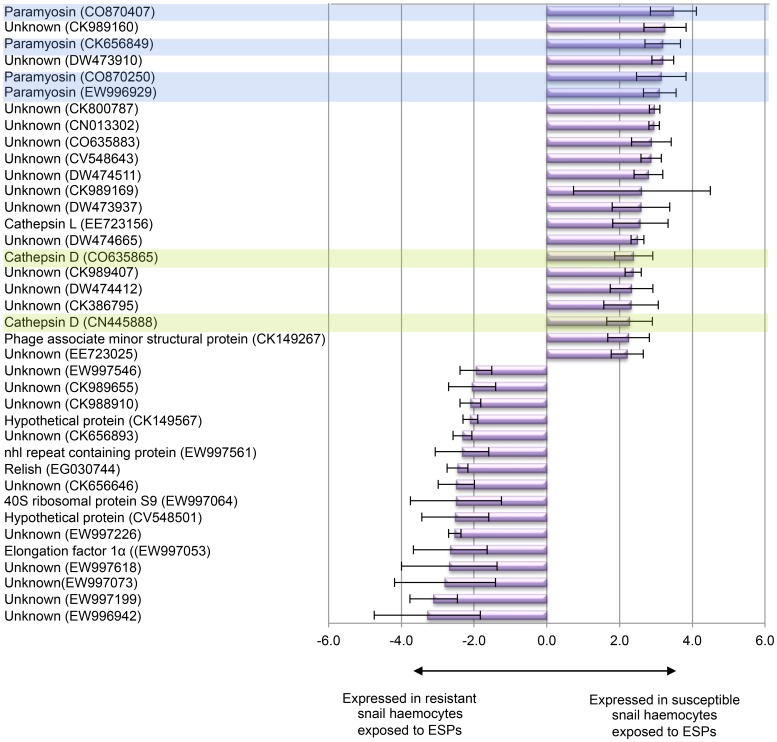
Genes differentially expressed following ESP exposure that are uniquely identified in this study. Comparative analysis of differentially expressed genes from this *in vitro* study with those from previous *in vivo* studies (Table S1) revealed 38 genes not previously identified as being involved in *S. mansoni*–*B. glabrata* interactions. Mean expression values (±SD) are shown for each individual differentially expressed gene (p≤0.05, n = 4). A positive value indicates the gene is expressed differentially in susceptible snail haemocytes whereas a negative value indicates the gene is expressed differentially in resistant snail haemocytes. Multiple genes matching the same protein are shaded in a similar colour.

Comparative analysis of differentially expressed haemocyte genes with those seen in our previous experiments performed following *in vivo* infection of *B. glabrata* with *S. mansoni* revealed: two unknown genes (DY523254/DY523255) previously identified in haemocytes 2–24 h post-infection by SSH [10]; 12 genes including elongation factor 2, ubiquitin-conjugating enzyme E2D 2, elastase 2-like, exported avidin family protein, one hypothetical protein; and a further seven unknowns found previously in haemocytes 2–24 h post-infection using the 2K microarray [24]. Additionally, 58 genes comprising 34 known, four hypothetical, and 20 unknown were in common with our most recent analysis 2 h post-exposure using the same 5K array [25], which explored gene expression between resistant and susceptible snail haemocytes when snails were either infected or not by *S. mansoni*. No genes were found in common with our previous differential display study [26]. Although a total of 60 genes were found differentially expressed in common with the other studies (Table S1), 38 were uniquely detected in the current study following 1 h ESP (20 μg/ml) exposure (Table S1; Fig. 3), comprising 16 resistant-specific and 22 susceptible-specific. The unique genes identified by BlastX that were resistant-specific were elongation factor 1α, 40S ribosomal protein S9, Relish, nhl repeat containing protein, whereas those susceptible-specific were phage associate minor structural protein, cathepsin D, cathepsin L, and paramyosin (Fig. 3).

## Discussion

Although cDNA microarrays [24], [25] and other techniques [10], [22], [23], [26] have been used to determine effects of *S. mansoni* on *B. glabrata* haemocyte gene expression profiles, including comparing between schistosome-resistant and -susceptible strains [24], [25], this is the first study to explore specifically gene expression patterns in haemocytes of these snails when exposed to *S. mansoni* larval transformation products. Defining haemocyte gene expression patterns in this way has permitted the interrogation of host differences when exposed to components released by the parasite during transformation, rather than those that might arise directly, or indirectly, in response to interactions between haemocytes and the schistosome surface. Ninety-eight genes were found differentially expressed representing 91 different proteins, with 57 genes being resistant-specific and 41 being susceptible-specific. Because haemocytes were exposed to ESPs for 1 h, we consider the differences to best represent those that might prevail during an immediate/early response in haemocytes, rather than those that would occur indirectly as a consequence of cellular feedback mechanisms.

The 60 differentially expressed genes found here that correspond with those identified in our previous studies following infection of *B. glabrata* with *S. mansoni* [10], [24]–[26] signify those that are either: 1) differentially expressed in the resistant or susceptible snail haemocytes before exposure and are thus constitutive phenotypic differences between the snail strains (discussed in [25]); or 2) expressed differentially (*in vitro* (here) or *in vivo* (other studies)) in response to ESPs (with a molecular weight of over 7,500) released by the transforming schistosome larvae. These genes include elastase-2, matrilin, elongation factor-2, ubiquitin conjugating enzyme E2D 2, and dermatopontin 2 in haemocytes from resistant snails, and 50S ribosomal protein I2, H-ras-like suppressor 3, neuromacin and theromacin in susceptible snail haemocytes. Dermatopontin is a cell adhesion molecule likely secreted by haemocytes [33] that might regulate haemocyte adhesion and encapsulation responses possibly with matrilin also playing a role. Dematopontin is upregulated in resistant *B. glabrata* exposed to *Echinostoma caproni* (as is matrilin) [33], and is downregulated at 0.5 days/upregulated at 1 day following infection of susceptible *B. glabrata* with *Echinostoma paraensei* [23] demonstrating the dynamic nature of dermatopontin gene expression during the course of infection. Differential expression in resistant snail haemocytes of the serine protease elastase 2 which degrades extracellular matrix molecules and can mediate bacterial killing [34] and of ubiquitin conjugating enzyme E2D 2 which marks misfolded/damaged proteins for degradation by the 26S proteasome [35] is indicative of increased proteolysis in the face of infection and parasite induced stress. In susceptible snail haemocytes the differential expression of antimicrobial peptides theromacin and neuromacin support an antimicrobial response [36], although this is insufficient to kill the invading schistosome larvae.

Importantly, the remaining 38 genes uniquely differentially expressed in the current study are most likely those expressed in response to the concentrations of ESPs (20 μg/ml) used, which were chosen to represent the conditions that might be experienced when haemocytes are in close proximity to transforming larvae [16], [18], [19]. This concentration of ESPs was shown by us to consistently down-regulate ERK signalling in schistosome-susceptible *B. glabrata* [18], with lower doses (1 μg/ml) resulting in reduced HSP70 protein expression in the same snail strain [19]. The uniquely differentially expressed genes should also include those expressed in response to ESPs that have the capacity to interact with haemocytes in the absence of lymph factors. Although we considered injecting ESPs into snails and subsequently collecting haemocytes, the quantity of ESP material generated during miracidium-to-mother-sporocyst development is very low and sufficient only for *in vitro* assays. Of these uniquely differentially expressed genes, the nuclear factor κB (NF-κB) subunit Relish was expressed by resistant snail haemocytes exposed to ESPs. In molluscs, as in other organisms [37], Relish is activated in response to immune challenge including by bacterial LPS in the abalone *Haliotis diversicolor* [38] and scallop *Chlamys farreri* [39]. Relish expression is also upregulated in the pearl oyster *Pinctata fucada* after injection of the bacterium *Vibrio alginolyticus* [40] and in the oyster *Crassostrea gigas* upon infection with *Vibrio caralliilyhus* [41]. In *B. glabrata*, Relish expression has recently been shown to increase 6 h post *S. mansoni* infection [42]. That Relish is differentially expressed in haemocytes from the different snail strains after 1 h ESP exposure implies that ESPs might induce early and specific effects on NF-κB signalling in haemocytes. Modulation of this transcription factor by *S. mansoni* is therefore worthy of further study because in humans and other organisms the NF-κB pathway is rapidly activated following infection resulting in expression of hundreds of early response genes including antimicrobial peptides [43]. Resistant-specific differential expression of elongation factor 1α, which binds t-RNA to ribosomes, and of 40S ribosomal protein S9 are indicative of increased protein translation in resistant snail haemocytes in general. On the other hand, differential expression of cathepsins D and L, intracellular lysosomal aspartic and cysteine proteinases respectively, by susceptible snail haemocytes in the presence of ESPs suggests an increased capacity to degrade protein, including that released by the parasite and internalized by the haemocyte. Because a cathepsin L-like protease precursor gene (CO870188) was found to be upregulated in resistant *B. glabrata* 2/24 h after snails were exposed to *S. mansoni* [24], it seems that intracellular haemocyte protease expression in response to *S. mansoni* is complex and might depend on the nature of the stimulus (e.g. ESPs verses intact parasites) and the duration of exposure. Twenty-four genes that displayed differential expression after 1 h ESP exposure and were unique to this study are of unknown function. It is anticipated that advances in functional genomics in snails, including gene knockdown with RNA interference [44], [45] to determine the function of some of these genes, will be critical in furthering our understanding of ESP-specific effects on haemocyte functional biology.

In our recently published work 417 genes (146 susceptible- and 271 resistant-specific) were differentially expressed in *B. glabrata* haemocytes following 2 h exposure to *S. mansoni in vivo* [25]. Therefore, as might be expected, the physical presence of the schistosome, possibly with its ciliated epidermal plates (which were removed from ESPs in the current study), appears to influence haemocyte gene expression profoundly, beyond that seen in response to ESPs alone (98 genes after 1 h). Genes for the molecular chaperone heat shock protein 70 (HSP70) have been found in many studies to be differentially expressed in *S. mansoni*-resistant snails in response to *S. mansoni* infection [10], [24], [25], [45], while in others they have been shown to be upregulated in the susceptible strain [46]. Given that HSP70 is a stress response protein that is activated rapidly by a plethora of potentially harmful stimuli [47], [48] it is perhaps surprising that when exposed to ESPs haemocytes from the different snail strains did not reveal differential HSP70 gene expression. When exposed to 20 μg/ml ESPs for 1 h, haemocyte HSP70 protein levels are significantly reduced in the same snail strains as those used in the current study partly through degradation by the proteasome [19], but recover only in the susceptible strain after 5 h. A possibility in the current study is that certain genes including HSP70 are constitutively expressed in the resistant strain more (or less) than in the susceptible, and that ESP dependent changes result in a situation where the mRNA levels become indistinguishable, masking any effect. However, in our recent analysis [25], constitutive expression of HSP70 did not differ between the same snail strains. Recently, HSPs were shown to play a role in *B. glabrata* resistance to *S. mansoni*, as exposing resistant *B. glabrata* to increased temperature for 4 h prior to *S. mansoni* infection resulted in a phenotype switch whereby the resistant snails became susceptible to the parasite [49]. Therefore it would be worthwhile studying HSP70 interaction (client) partners in *B. glabrata* to better understand how the dynamics of HSP gene and protein expression might influence cellular outcome in the face of schistosome infection.

When evaluating the effects of schistosomes/schistosome components on snail defence cell gene expression, it is valuable to consider also how such effects might initially be mediated. It has been shown that the activity of the ERK pathway is suppressed by ESPs in haemocytes from schistosome susceptible snails but not in those from the resistant strain [18]. One transcription factor that is likely activated by ERK in *B. glabrata* is Elk-1 [50] and in humans this transcription factor appears to target genes that are involved in expression control, including basal transcriptional machinery components and the spliceosome and ribosome [51]. Thus the reduced expression of certain susceptible-specific genes both here and in other studies may be due to the attenuation of ERK signalling in susceptible snail haemocytes.

Despite multiple gene expression studies there is still much to learn concerning the schistosome-snail host-parasite relationship and the nature of resistance to the parasite. It is clear, however, that schistosome ESPs produced during early parasite development do affect host snail haemocytes. Investigations into the effects of the ciliary epidermal plates that are shed rapidly upon snail invasion on haemocyte physiology and gene expression patterns are also needed. Through integration of gene expression studies and functional biology it is hoped that we will arrive at a more complete understanding of *B. glabrata-S. mansoni* molecular interactions, a necessary prerequisite to the design of schistosomiasis control strategies, which challenge successful infections in natural populations.

## Supporting Information

Table S1 **Differentially expressed genes.**
(DOCX)
