# Supplementary material for: Differences in the Gene Expression Profiles of Haemocytes from Schistosome-Susceptible and -Resistant Biomphalaria glabrata Exposed to Schistosoma mansoni Excretory-Secretory Products
Source: PLoS One. 2014 Mar 24;9(3):e93215. doi: 10.1371/journal.pone.0093215 (PMC3963999; doi:10.1371/journal.pone.0093215)
Supplement: Table S1 — Differentially expressed genes. (DOCX) [file pone.0093215.s001.docx]

**Table S1. Differentially expressed genes.**

| GenBank Accession No. | Source**^1^** | Mean Value | SD of 4 arrays | BLASTX match | E value | Organism | SSH**^2^** [10] | M1**^2^**  [23] | M2**^2,3^**  [24] |
| --- | --- | --- | --- | --- | --- | --- | --- | --- | --- |
|  |  |  |  |  |  |  |  |  |  |
| EW997087 | SSH | -5.273 | 0.415 | Hypothetical protein | 1.34455E-18 | *Vittaforma corneae* |  |  | *RB* |
| EW997105 | SSH | -4.994 | 0.412 | Exported avidin family protein | 6.85296E-23 | *Burkholderia glumae* |  |  |  |
| CV548474 | ORESTES | -4.915 | 1.008 | Hypothetical protein | 2.75586E-15 | *Vittaforma corneae* |  |  | *RB* |
| CO870321 | ORESTES | -3.812 | 0.586 | Unknown |  |  |  |  | *RB* |
| EW997096 | SSH | -3.580 | 0.378 | Unknown |  |  |  |  | *RC* |
| EW996942 | SSH | -3.288 | 1.459 | Unknown |  |  |  |  |  |
| CK149390 | ORESTES | -3.143 | 0.566 | Unknown |  |  |  |  | *RB* |
| EW997449-**c** | SSH | -3.138 | 0.707 | Unknown |  |  |  |  | *RE* |
| EW997199 | SSH | -3.115 | 0.655 | Unknown |  |  |  |  |  |
| EW996827 | SSH | -3.071 | 0.450 | Elastase 2-like EC 3.4.21.0 | 1.2518E-28 | *Danio rerio* |  |  | *RB* |
| EW996998-**b** | SSH | -3.067 | 0.603 | Unknown |  |  |  |  | *RB/C* |
| EW996825-**c** | SSH | -3.060 | 0.744 | Unknown |  |  |  |  | *RE* |
| CV548443 | ORESTES | -3.047 | 1.333 | Unknown |  |  |  |  | *RE* |
| EW997106-**c** | SSH | -3.012 | 0.799 | Unknown |  |  |  |  | *RE* |
| CV548492 | ORESTES | -2.976 | 1.409 | Unknown |  |  |  |  | *RE* |
| EW997485-**b** | SSH | -2.959 | 0.808 | Unknown |  |  |  |  | *RB/C* |
| CV548460 | ORESTES | -2.955 | 1.865 | Unknown |  |  |  |  |  |
| DY523254-**b** | SSH | -2.937 | 0.760 | Unknown |  |  |  |  | *RB/C* |
| EW997073 | SSH | -2.801 | 1.389 | Unknown |  |  |  |  |  |
| EW997397 | SSH | -2.761 | 0.258 | Hypothetical protein | 1.26243E-05 | *Hydra magnipapillata* |  |  | *RC* |
| EW997560 | SSH | -2.724 | 0.914 | Nas-14 protein EC 3.4.24.0 | 9E-11 | *Caenorhabditis elegans* |  |  | *RB* |
| EW997618 | SSH | -2.681 | 1.312 | Unknown |  |  |  |  |  |
| EW997053 | SSH | -2.654 | 1.013 | Elongation factor 1α | 5.96077E-15 | *Beauveria bassiana* |  |  |  |
| CO870356 | ORESTES | -2.623 | 0.776 | Unknown |  |  |  |  | *RB* |
| CK656770-**d** | ORESTES | -2.592 | 0.895 | Matrilin | 6.30855E-34 | *Biomphalaria glabrata* |  |  | *RB/C* |
| EW997445 | SSH | -2.566 | 0.252 | Unknown |  |  |  |  | *RB* |
| DY523255 | SSH | -2.565 | 0.661 | Unknown |  |  |  |  | *RB* |
| CK656733 | ORESTES | -2.560 | 0.697 | Ubiquitin-conjugating enzyme E2D 2  EC 6.3.2.19 | 2.57508E-20 | *Rattus norvegicus* |  |  | *RE* |
| EW997555-**e** | SSH | -2.549 | 0.933 | Elongation factor-2 | 7E-32 | *Mus musculus* |  |  | *RE* |
| EW997226 | SSH | -2.532 | 0.171 | Unknown |  |  |  |  |  |
| CV548501 | ORESTES | -2.518 | 0.922 | Hypothetical protein | 1.33041E-06 | *Nematostella vectensis* |  |  |  |
| EW997067-**e** | SSH | -2.517 | 0.954 | Elongation factor-2 | 2.05E-06 | *Salmo salar* |  |  | *RE* |
| EW997064 | SSH | -2.499 | 1.251 | 40S ribosomal protein S9 | 2.63106E-60 | *Capitella teleta* |  |  |  |
| CK656646 | ORESTES | -2.490 | 0.501 | Unknown |  |  |  |  |  |
| EW997424 | SSH | -2.484 | 0.969 | Unknown |  |  |  |  | *RE* |
| EW997345 | SSH | -2.481 | 0.368 | Unknown |  |  |  |  | *RB* |
| EG030744 | ORESTES | -2.458 | 0.287 | Relish (nuclear factor nf-kappa-b p105 subunit-like) | 1.46829E-62 | *Biomphalaria glabrata* |  |  |  |
| CK988725-**d** | EST | -2.378 | 0.933 | Matrilin | 2.44E-104 | *Biomphalaria glabrata* |  |  | *RB/C* |
| EW997561 | SSH | -2.334 | 0.742 | nhl repeat containing protein | 1.10069E-34 | *Fibrisoma limi* |  |  |  |
| CK656893 | ORESTES | -2.320 | 0.258 | Unknown |  |  |  |  |  |
| EW997432 | SSH | -2.311 | 0.910 | Unknown |  |  |  |  | *RE* |
| CK988686 | EST | -2.308 | 0.347 | Dermatopontin 2 | 1.66008E-63 | *Biomphalaria glabrata* |  |  | *RB* |
| EW997342 | SSH | -2.287 | 1.035 | Actin | 6.03758E-63 | *Brachionus plicatilis* |  |  | *RE* |
| CV548469 | ORESTES | -2.259 | 1.060 | Unknown |  |  |  |  | *RE* |
| CK988741 | EST | -2.179 | 0.379 | Dermatopontin 2 | 3.26784E-49 | *Biomphalaria glabrata* |  |  | *RC* |
| EW997374-**a** | SSH | -2.108 | 0.561 | Unknown |  |  |  |  | *RB* |
| CK149567 | ORESTES | -2.106 | 0.205 | Hypothetical protein | 1E-27 | *Magnetospirillum gryphiswaldense* |  |  |  |
| CK988910 | EST | -2.102 | 0.285 | Unknown |  |  |  |  |  |
| CV548449 | ORESTES | -2.097 | 0.208 | Tetratricopeptide tpr_2 | 7.32406E-33 | *Philodina roseola* |  |  | *RE* |
| DY523259-**a** | SSH | -2.087 | 0.614 | Unknown |  |  |  |  | *RB* |
| CK989655 | EST | -2.054 | 0.646 | Unknown |  |  |  |  |  |
| CV042579 | EST | -2.052 | 0.811 | Unknown |  |  |  |  | *RC* |
| CK656903 | ORESTES | -2.051 | 0.671 | Unknown |  |  |  |  | *RB* |
| CK656890 | ORESTES | -2.045 | 0.184 | Unknown |  |  |  |  | *RB* |
| CK989609 | EST | -1.980 | 0.484 | Unknown |  |  |  |  | *SB* |
| CV548156 | ORESTES | -1.968 | 0.447 | Neural ectodermal development factor imp-l2 | 5.67217E-29 | *Crassostrea gigas* |  |  | *RC* |
| EW997546 | SSH | -1.951 | 0.439 | Unknown |  |  |  |  |  |
| CK989051 | EST | 1.968 | 0.263 | Hypothetical protein | 0.00000007 | *Xenopus (Silurana) tropicalis* |  |  | *SB* |
| EE723025 | EST | 2.211 | 0.440 | Unknown |  |  |  |  |  |
| DW474522 | EST | 2.244 | 0.335 | Unknown |  |  |  |  | *SE* |
| CK149267 | ORESTES | 2.247 | 0.571 | Phage associate minor structural protein | 2.11092E-09 | *Nematostella vectensis* |  |  |  |
| CN445888 | EST | 2.276 | 0.628 | Cathepsin D EC 3.4.23.0 | 3E-33 | *Todarodes pacificus* |  |  |  |
| EE723175 | EST | 2.316 | 0.659 | 50s ribosomal protein l2 | 2.4848E-07 | *Ixodes scapularis* |  |  | *SB* |
| CK386795 | EST | 2.316 | 0.748 | Unknown |  |  |  |  |  |
| DW474747 | EST | 2.328 | 0.240 | Unknown |  |  |  |  | *SB* |
| DW474412 | EST | 2.328 | 0.588 | Unknown |  |  |  |  |  |
| CK989407 | EST | 2.377 | 0.219 | Unknown |  |  |  |  |  |
| CO635865 | EST | 2.391 | 0.523 | Cathepsin D EC 3.4.23.0 | 3.7623E-44 | *Capitella teleta* |  |  |  |
| DW474665 | EST | 2.495 | 0.173 | Unknown |  |  |  |  |  |
| CV548309 | ORESTES | 2.535 | 0.431 | Unknown |  |  |  |  | *SB* |
| EE723156 | EST | 2.569 | 0.764 | Cathepsin L | 1.5504E-32 | *Haliotis discus* |  |  |  |
| DW474845 | EST | 2.584 | 1.110 | Hras-like suppressor 3 | 1E-17 | *Perca flavescens* |  |  | *SB* |
| DW473937 | EST | 2.594 | 0.792 | Unknown |  |  |  |  |  |
| CK988781 | EST | 2.595 | 0.479 | Unknown |  |  |  |  | *SB* |
| CK989169 | EST | 2.611 | 1.884 | Unknown |  |  |  |  |  |
| CO870292 | ORESTES | 2.619 | 1.149 | Unknown |  |  |  |  | *SB* |
| EW996739 | SSH | 2.668 | 0.236 | ATP synthase subunit b | 6.85965E-13 | *Cerebratulus lacteus* |  |  | *SB* |
| EW996804 | SSH | 2.681 | 0.264 | Unknown |  |  |  |  | *SB* |
| CV548134 | ORESTES | 2.744 | 0.511 | Unknown |  |  |  |  | *SB* |
| DW474511 | EST | 2.795 | 0.398 | Unknown |  |  |  |  |  |
| EW996791 | SSH | 2.854 | 0.314 | Unknown |  |  |  |  | *SB* |
| CV548643 | ORESTES | 2.870 | 0.279 | Unknown |  |  |  |  |  |
| CO635883 | EST | 2.876 | 0.550 | Unknown |  |  |  |  |  |
| CN013302 | EST | 2.953 | 0.142 | Unknown |  |  |  |  |  |
| CK800787 | EST | 2.964 | 0.142 | Unknown |  |  |  |  |  |
| CK989034 | EST | 2.992 | 0.400 | Cytidine deaminase EC 3.5.4.5 | 2.41365E-96 | *Biomphalaria glabrata* |  |  | *SE* |
| EW996929 | SSH | 3.102 | 0.452 | Paramyosin | 4.29E-32 | *Crassostrea gigas* |  |  |  |
| CO870250-**f** | ORESTES | 3.146 | 0.681 | Paramyosin | 9.03E-71 | *Haliotis discus* |  |  |  |
| DW473910 | EST | 3.188 | 0.300 | Unknown |  |  |  |  |  |
| CK656849 | ORESTES | 3.188 | 0.486 | Paramyosin | 1.8552E-06 | *Mytilus galloprovincialis* |  |  |  |
| CK989160 | EST | 3.254 | 0.579 | Unknown |  |  |  |  |  |
| CK327222 | EST | 3.399 | 0.568 | Unknown |  |  |  |  | *SB* |
| CO870407-**f** | ORESTES | 3.483 | 0.629 | Paramyosin | 3.69E-41 | *Crassostrea gigas* |  |  |  |
| CK989482 | EST | 3.589 | 0.405 | Neuromacin-like protein | 9.37157E-10 | *Aplysia californica* |  |  | *SB* |
| CK989131 | EST | 3.657 | 0.521 | Theromacin | 9.00799E-16 | *Hyriopsis cumingii* |  |  | *SB* |
| CK988968 | EST | 4.191 | 0.572 | Unknown |  |  |  |  | *SB* |
| CV548292 | ORESTES | 4.435 | 0.766 | Unknown |  |  |  |  | *SB* |
| CO654043 | EST | 4.967 | 0.331 | Unknown |  |  |  |  | *SB* |

Genes identified as being differentially expressed between haemocytes from *S. mansoni*-resistant and –susceptible *B. glabrata* when exposed to excretory-secretory products for 1 h, ranked in order of mean (n=4) fold difference in expression, based on normalized intensity ratios. Negative values indicate clones that are resistant associated whereas positive values show those that are susceptible associated.

**a-f** Clusters of overlapping sequences

**^1^**Source indicates whether the clone was derived from suppression subtractive hybridization (SSH) library, expressed sequence tag (EST) libraray, or open reading frame EST library.

**^2^**Differentially expressed genes also found in the following suppression subtractive hybridization (SSH), or microarray (M1, 2008; M2, 2012) studies are highlighted:

10. Lockyer AE, Spinks J, Noble LR, Rollinson D, Jones CS (2007) Identification of genes involved in interactions between *Biomphalaria glabrata* and *Schistosoma mansoni* by suppression subtractive hybridization. Molecular & Biochemical Parasitology 151: 18–27.

23. Lockyer AE, Spinks J, Kane RA, Hoffmann KF, Fitzpatrick JM, et al. (2008) *Biomphalaria glabrata* transcriptome: cDNA microarray profiling identifies resistant- and susceptible-specific gene expression in haemocytes from snail strains exposed to *Schistosoma mansoni*. BMC Genomics 9: 634.

24. Lockyer AE, Emery AM, Kane R a, Walker AJ, Mayer CD, et al. (2012) Early differential gene expression in haemocytes from resistant and susceptible *Biomphalaria glabrata* strains in response to *Schistosoma mansoni*. PloS One 7: e51102.

**^3^**Notations under M2 indicate whether the gene was found differentially expressed in RC (resistant control), RE (resistant exposed), or RB (resistant both control and exposed), or SC, SE, SB – susceptible, respectively. Genes identified as differentially expressed in both control and exposed indicate strain specific differences.
